# Early-Term Effect of Bilateral Sequential Cochlear Implantation on the Audiovestibular Function of Paediatric Patients: A Prospective Pilot Study

**DOI:** 10.3390/brainsci16060579

**Published:** 2026-05-29

**Authors:** Federica Di Berardino, Marco Pozzi, Anna Maria Gasbarre, Valeria Castelli, Cinzia Lazzarini, Giorgio Lilli, Diego Zanetti, Valerio Maria Di Pasquale Fiasca

**Affiliations:** 1 Department of Clinical Sciences and Community Health, University of Milan, 20122 Milan, Italy; 2 Audiology Unit, Department of Surgical Sciences, Foundation I.R.C.C.S. Ca’ Granda Ospedale Maggiore Policlinico, 20122 Milan, Italy

**Keywords:** sequential cochlear implant, children, balance, vestibular function

## Abstract

**Highlights:**

**What are the main findings?**

- Sequential bilateral cochlear implantation significantly improved unilateral speech recognition with the second implant over the first 12 months after activation.
- Speech perception in noise improved significantly, while the vestibular function remained stable overall without evidence of major short-term deterioration.

**What are the implications of the main findings?**

- Sequential bilateral cochlear implantation appears to provide meaningful early auditory benefit in paediatric patients, particularly for the second-implant performance and listening in noisy environments.
- These findings support the clinical safety and effectiveness of sequential bilateral implantation, although larger studies are needed to confirm long-term audiological and vestibular outcomes.

**Abstract:**

Background/Objectives: Sequential bilateral cochlear implantation is being increasingly considered in paediatric patients with bilateral profound hearing loss, particularly when the contralateral ear shows limited residual hearing or poor speech discrimination. Although auditory benefits of a second cochlear implant (CI2) have been reported, concerns remain regarding possible vestibular impairment after surgery. This study aimed to provide a preliminary prospective evaluation of early audiological and vestibular outcomes in children undergoing sequential bilateral cochlear implantation and to explore whether the age at implantation or the inter-implant interval influenced longitudinal trajectories. Methods: Ten children with prelingual bilateral profound sensorineural hearing loss who underwent sequential bilateral cochlear implantation were prospectively enrolled. Assessments were performed at baseline and during follow-up up to 12 months after CI2 activation. The audiological evaluation included pure-tone audiometry, speech recognition in quiet with CI2 alone and bilaterally, and speech-in-noise testing in frontal and lateralised configurations. The vestibular evaluation included static stabilometry and video head impulse testing. Patient-reported outcomes were assessed using the speech, spatial and qualities of hearing scale for children. Longitudinal changes were analysed using mixed-effects models with false discovery rate correction. Results: Unilateral speech recognition with CI2 improved significantly over time, with median word recognition increasing from 0% at 3 months to 60% at 12 months (*p* < 0.0001; q = 0.0001). The speech-in-noise performance also improved significantly in the frontal and F1C configurations. Bilateral speech recognition in quiet remained stable at high levels, consistent with a ceiling effect. The vestibular measures were broadly stable at the group level, and the SSQ scores showed modest, non-significant changes. Exploratory analyses did not identify consistent effects of the implantation age or inter-implant interval on longitudinal trajectories. Conclusions: Sequential bilateral cochlear implantation was associated with early auditory benefit, particularly for CI2 unilateral performance and speech perception in noise, without evidence of major short-term vestibular deterioration.

## 1. Introduction

Since its introduction in clinical practice, the cochlear implant (CI) has revolutionised the audiological rehabilitation of paediatric patients. In children with bilaterally severe-to-profound hearing loss, a second implant can provide significant advantages in sound localisation, language development, and word discrimination in noise [1,2,3,4]. Therefore, bilateral cochlear implantation is currently widely practised and is regarded as an optimal option [5]. The intervention is recommended to be initiated at an early age to maximise the likelihood of hearing rehabilitation and language development [6,7,8]. The patient may receive the devices during a single surgical procedure or sequentially. Simultaneous positioning of the CIs represents the best rehabilitative option for bilaterally deaf children [9,10].

In unilaterally implanted children with poor residual hearing or poor discrimination skills in the contralateral ear, CIs may be applied sequentially to optimise audiological outcomes [8,11,12]. Several studies have examined the optimal timing of CI2 placement, which is generally recommended to be short to maximise hearing capacity [10,13,14] and to optimise use of the second device [15]. Nonetheless, improvement was also observed with longer inter-implantation intervals [16,17]. Better audiological outcomes were observed in patients who also had CI2 implanted before age 5 [18].

The surgical positioning of the array within the internal ear may influence the vestibular function. This finding has already been reported in the adult population [19,20,21,22] and children [23]. The same risk of vestibular dysfunction could apply to children undergoing CIs [24], especially in bilateral simultaneous implants [25]. In paediatric patients, a saccular affection was evidenced postoperatively by an increase in vestibular evoked myogenic potential (VEMP) abnormal responses [26,27,28]. Conversely, similar findings were not observed for caloric testing, whereas sufficient data were not available for the video head impulse test (vHIT) and posturography. Furthermore, the literature provides no evidence of subjective symptoms, such as dizziness. The authors generally reported a lack of data and studies on this aspect [27], and subsequent studies have refuted this association [29,30,31]. Potentially, the vestibular affection could cause clinical manifestations such as postoperative imbalance, vertigo, and falls, and alterations in vHIT and other tests results. The possible mechanisms leading to this complication include direct trauma related to insertion of the electrode, intraoperative perilymphatic fluid leakage, electrical stimulation of the otolithic organs, and foreign body labyrinthitis [32]. Furthermore, other factors such as the aetiology of the hearing loss [33] and the characteristics of the surgical procedure [34] could influence the risk of postoperative vestibular affection. Therefore, while aiming to ensure the optimal audiological outcomes, children with unilateral implantation should undergo careful assessment, including an evaluation of vestibular function, before receiving a CI2 [26,35,36,37].

The aim of the present study was to provide a preliminary evaluation of the audiological and vestibular early-term outcomes in a selected cohort of paediatric patients who underwent sequential bilateral CIs. We tested whether vestibular function may be preserved in the early term and whether any associations with auditory function are detectable. In this analysis, vestibular organ activity and the balance function were assessed using static and dynamic posturography.

## 2. Materials and Methods

In the present study, we enrolled a cohort of paediatric patients, ranging between 7 and 15 years of age, at the CI2. All the patients received a diagnosis of prelingual bilateral sensorineural profound hearing loss. All these subjects underwent bilateral sequential CIs. All the patients received a perimodiolar array. No complications in positioning, including intracochlear trauma, scala shift, and tip fold-over, were reported intraoperatively. Postoperative imaging (cone beam ear CT without contrast) further excluded complications in the array positioning. We excluded patients with single-sided deafness, cognitive impairment, anomalous cochlear morphology, complications during the surgical procedure—particularly during the array positioning phase—and reimplantation cases. This study received approval by the Ethical Committee of Fondazione IRCCS Ca’ Granda Ospedale Maggiore Policlinico of Milan, Italy (IRB N8 403_2017Bilaterals, 10 July 2017).

The study was structured to include a baseline assessment at enrolment and postoperative follow-up evaluations at 3, 6, 9, and 12 months, as outlined in Table 1.

The patients underwent a battery of audiological and vestibular exams. The hearing was assessed through the following tests: pure-tone air-conduction audiometry (with headphones); speech audiometry under quiet open-field conditions, using both the CIs (bilateral) and only the CI2 (unilateral); and speech-in-noise audiometry. Pure-tone audiometry was performed using a Natus Aurical Aud audiometer (Otometrics-Natus Medical, Pleasanton, CA) with headphones (Telephonics TDH-39P) in a quiet room (bilateral noise level, <40 dB HL; room reverberation time, 0.41 s). Speech audiometry in quiet was carried out in the sound field using a Natus Aurical audiometer and Amplaid loudspeakers, employing Rossi–Rimondini speech materials. Speech-in-noise testing was conducted in the sound field using the SRT50 protocol, an adaptive procedure in which the signal-to-noise ratio (SNR) is adjusted by 2 dB for each word based on the participant’s performance. The speech signal consisted of bisyllabic words (OTOsuite word list) presented at a fixed level of 65 dB HL, while the competing noise (babble noise) was initially set at 45 dB HL. The outcome measure was the SRT50% SNR. Testing was administered in three spatial configurations:

FF: speech and noise both from the front at a 1 m distance;

F1C: speech from the front and noise from the side of the 1st CI (90° angle);

F2C: speech from the front and noise from the side of the CI2 (90° angle).

Across all speech tests, the same word lists, equipment, and test room were used to keep presentation levels constant at each session, thereby ensuring the reliability and comparability of the outcomes [38].

The vestibular tests included: static stabilometry [39] and vHIT. The following questionnaires were administered: the speech, spatial, and qualities (SSQ) questionnaires for children [40] and the Italian language evaluation battery for the assessment of emotional and linguistic prosody, administered by a specialised speech-language pathologist with expertise in deafness and hearing impairment [41].

Static stabilometry (S.V.E.P. force platform; Politecnica, Modena, Italy) was performed with 52 s trials under four conditions: eyes open, eyes closed, eyes open on a foam pad, and eyes closed on a foam pad (Orsafoam, Gorla Minore, Italy). The outcomes included the path length, sway area, and sway velocity. A sensory analysis was also provided, expressing as percentages the contribution of each sensory system to the balance control [42]. The vHIT was recorded using the Natus ICS Impulse system. It was performed using passive, rapid, small-amplitude head impulses while the child fixated on a stationary target at 1.1 m in front of the patient. Vestibulo-ocular reflex gain and corrective saccades were recorded and analysed.

For the present study, the function of the lateral semicircular canals was assessed. Gain and asymmetry were analysed. Administration of the questionnaires and the language evaluation battery assessment was always performed by the same examiner.

### Statistical Analysis

All statistical analyses were performed using R (R Foundation for Statistical Computing, Vienna, Austria) [43]. Longitudinal outcomes were analysed with mixed-effects models to account for repeated measurements within each child. For each endpoint, we modelled the outcome over time (months from CI2 activation) with a subject-specific random intercept. We assessed longitudinal change using linear mixed-effects models with a subject-specific random intercept to account for repeated measures. Time was modelled as a categorical factor, and the global effect of time was tested via likelihood ratio tests (LRTs) comparing the time model against a null (intercept-only) model. To address multiplicity across outcomes, *p*-values from the global time tests were adjusted using a false discovery rate (Benjamini–Hochberg) correction, and the results are reported as nominal *p*-values and FDR-adjusted q-values.

To evaluate whether the trajectory over time differed according to (i) the age at first implantation (CI1 age), (ii) the age at second implantation (CI2 age), and (iii) the inter-implant interval, we fitted separate interaction models, including a time × predictor term (predictors were analysed as continuous variables, not as subgroups). The results are presented as: the Δ slope per +1 year, i.e., how much the monthly rate of change (improvement or worsening) varies for each additional year of age/interval, and the Δ change over follow-up per +1 year, which translates the same effect into the expected difference in the total change over the follow-up window (12 months for audiological/SSQ outcomes; 6 months for vestibular outcomes). For example, for speech recognition (% correct), a Δ change over follow-up per +1 year = −8 percentage points means that an additional year of age is associated with ~8 percentage points less improvement over follow-up. Given the small sample size, analyses were interpreted as hypothesis-generating. Effect estimates are reported with 95% confidence intervals, and multiplicity across the interaction tests was addressed using a false discovery rate (FDR) correction.

## 3. Results

In this analysis, 10 patients (5 F; 5 M) were enrolled. The median age of the patients was 10.5 years, ranging from 7 to 15 years (IQR = 2.75 years). The median age at first cochlear implantation (CI1) was 2 years, ranging from 2 to 3.5 years (IQR = 1.5 years). Importantly, three children in the sample underwent surgery for CI1 after the age of 3 years, specifically at 4, 6, and 7 years, whereas one child underwent implantation at 4 months of age. The median time interval between the CI1 and the CI2 was 8 years, with a maximum of approximately 15 years and a minimum of approximately 1.5 years (IQR = 4.25 years). Surgery for CI2 was performed, on average, 2 months after enrolment. The median age at CI2 was 10.5 years, ranging from 10 to 12.5 years (IQR = 2.75 years). All the demographic data are reported in Table 2.

### 3.1. Audiological Outcomes

**Figure 1:**
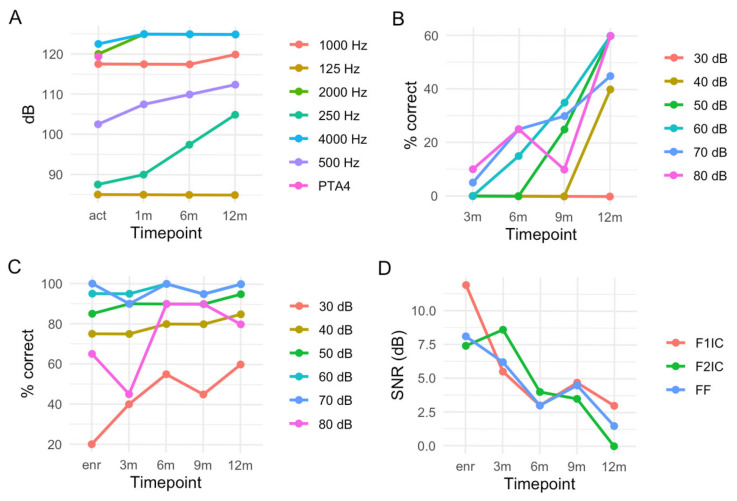
Hearing outcomes. (**A**) Unaided hearing threshold at 125, 250, 500, 1000, 2000 and 4000 Hz. (**B**) Word recognition score progression over time using only the CI2. (**C**) Word recognition score progression over time using both the CIs. (**D**) Results of the tests in noise.

The audiological longitudinal outcomes are summarised in Table 3 and Figure 1. Overall, the pure tone average (average of hearing threshold in dB nHL at 0.5, 1, 2 and 4 kHz; PTA-4) values were very high at all post-activation timepoints, with the median PTA4 increasing slightly from activation to 12 months (median: 115.6 dB at activation to 120.0 dB at 12 months) and a narrow interquartile range across follow-up. Speech recognition under the bilateral condition at 60 dB (word recognition score, WRS) was already high at enrolment (median: 95%, IQR: 80–100) and remained consistently high over follow-up, with median values between 95% and 100% at all timepoints, suggesting a ceiling effect for this outcome (Table 3). In contrast, speech recognition under the unilateral CI2 condition showed a marked improvement over time: the median WRS was 0% at 3 months (IQR: 0–7.5), increased to 15% at 6 months (IQR: 0–55) and 35% at 9 months (IQR: 12.5–75), and reached 60% at 12 months (IQR: 32.5–87.5), indicating progressive acquisition of speech recognition with the second implant. For the speech-in-noise performance (SRT50 expressed as SNR dB; more negative values indicate better performance), the median SRT50 improved over follow-up across conditions. In the frontal configuration (FF), the median SRT50 decreased from 8.1 dB at enrolment (IQR: 6.7–10.4) to 1.5 dB at 12 months (IQR: −3.0 to 4.5). Similar downward trends were observed in both the spatial configurations F1C and F2IC, with the lowest median SRT50 at 12 months for the F2IC condition (median: 0.0 dB, IQR: −0.75 to 1.45) (Table 3).

### 3.2. Vestibular Outcomes

**Figure 2:**
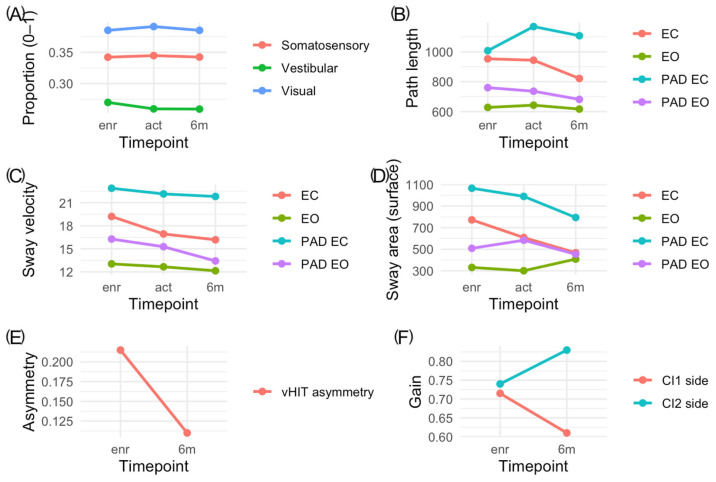
Vestibular assessment results. EO: eyes open; EC: eyes closed; PAD EC: foam surface with eyes open; PAD EO: foam surface with eyes closed.

The vestibular outcomes are reported in Table 4 and Figure 2. The sensory analysis derived from stabilometry (S.V.E.P.) showed broadly stable distributions across timepoints. The median somatosensory contribution remained approximately 0.34 at enrolment, activation, and 6 months, while the median vestibular contribution was ~0.27 at enrolment and ~0.26 at activation and 6 months. The median visual contribution was ~0.39 at enrolment and activation and ~0.39 at 6 months. For vHIT measures, data were available for eight participants at enrolment and nine at 6 months. The absence of a case at enrolment was caused by the patient’s non-cooperation due to a very young age. The median vHIT asymmetry decreased from 0.215 at enrolment (IQR: 0.048–0.428) to 0.110 at 6 months (IQR: 0.060–0.270), while the median gain on the CI2 side increased from 0.740 (IQR: 0.538–0.870) to 0.830 (IQR: 0.640–0.960). Given the limited sample size and incomplete vHIT data at both timepoints, these findings should be interpreted descriptively.

### 3.3. Patient-Reported Outcomes (SSQ)

**Figure 3:**
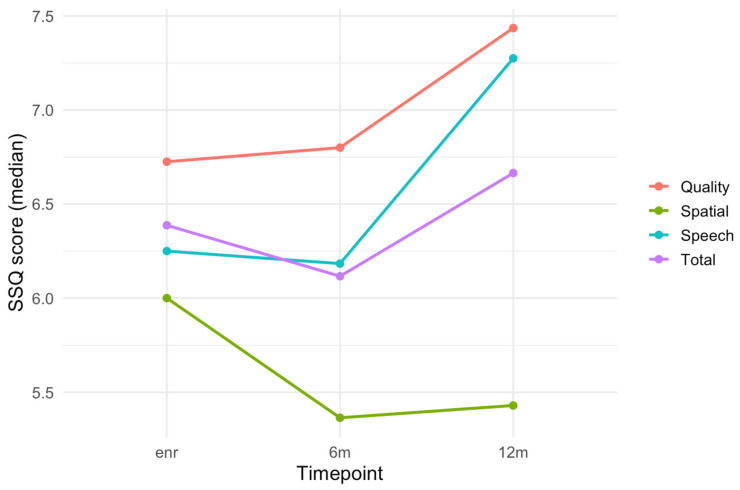
SSQ questionnaire results.

The SSQ domain and total scores are shown in Table 5 and Figure 3. The SSQ total scores were stable from enrolment to 6 months (median: 6.39 at enrolment vs. 6.12 at 6 months), with a modest increase by 12 months (median: 6.66, IQR: 6.42–6.78). A similar pattern was observed in the SSQ speech, which increased from a median of 6.25 at enrolment to 7.28 at 12 months. The SSQ quality also increased by 12 months (median: 7.44), whereas the SSQ spatial scores showed a slight decrease from enrolment to 6 months and remained similar at 12 months (Table 5). Overall, the SSQ results suggest modest improvements in perceived listening abilities over the first postoperative year, with domain-specific heterogeneity. 

### 3.4. Linguistic Test Battery

**Figure 4:**
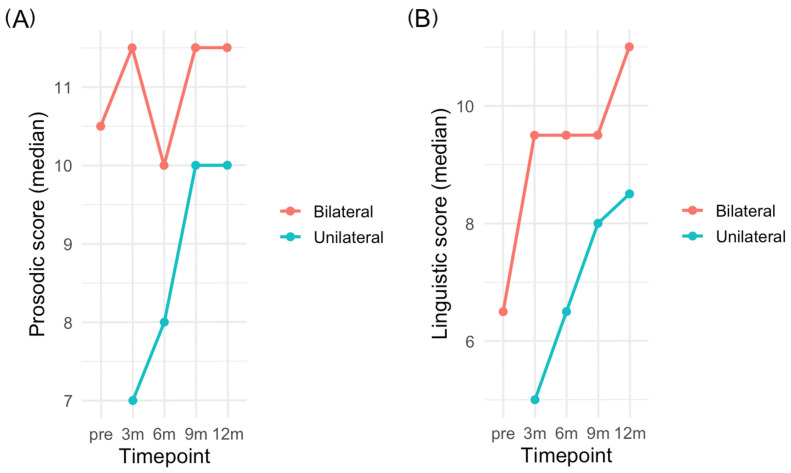
Linguistic test battery results.

In the linguistic domain, the median scores were consistently higher in the bilateral group than in the unilateral group across follow-up. In the bilateral group, the linguistic scores increased from 6.50 [5.00–8.00] pre-surgery to 9.50 [8.00–11.75] at 3 months and further to 11.00 [10.00–12.00] at 12 months. In the unilateral group, the linguistic scores rose from 5.00 [4.00–6.75] at 3 months to 8.50 [6.00–9.75] at 12 months, indicating a slower improvement over time. In the prosodic domain, the bilateral group showed relatively high scores already at pre-surgery (10.50 [7.25–11.75]) and maintained a stable performance over time, with median values of 11.50 [11.00–12.00] at both 9 and 12 months. The unilateral group showed a progressive increase in the prosodic scores, from 7.00 [5.00–9.00] at 3 months to 10.00 [9.00–10.75] at 12 months. Overall, both groups improved over time, but the bilateral group showed higher and more stable median scores, particularly for the linguistic performance. The results are depicted in Figure 4.

### 3.5. Change over Time

To evaluate whether the outcomes changed over follow-up, we fitted time-only mixed-effects models with time treated as a categorical factor and tested the global contribution of time using LRTs, with an FDR correction across outcomes. A significant overall time effect was observed for unilateral CI2 speech recognition at 60 dB (speech audiometry with unilateral CI at 60 dB; LRT *p* < 0.0001, FDR q = 0.0001), indicating an improvement over time. Likewise, the speech-in-noise performance showed significant time effects in the FF configuration (SRT50 FF; *p* = 0.0003, q = 0.0014) and in the F1C configuration (SRT50 F1C; *p* < 0.0001, q < 0.0001), consistent with overall improvement in noise across follow-up. For SRT50 F2IC, the global time effect was nominally significant (*p* = 0.024), but did not remain significant after FDR correction (q = 0.072), and should therefore be interpreted cautiously. PTA4 showed a borderline nominal time effect (*p* = 0.046) that did not survive FDR adjustment (q = 0.111), suggesting no strong evidence of systematic change over time at the group level once multiplicity was considered. No significant time effects were detected for bilateral speech recognition in quiet (speech audiometry with bilateral CIs at 60 dB; *p* = 0.618, q = 0.618), consistent with ceiling-level performance across timepoints. Similarly, the SSQ domain and total scores did not show significant global time effects (all q ≈ 0.27–0.57), and vHIT gain/asymmetry did not show significant change over the available follow-up (all q ≥ 0.47), noting that vHIT outcomes were available at only two timepoints and in nine participants. Results are reported in Table 6.

### 3.6. Effect Modification by Age at Implantation and Inter-Implant Interval

To explore whether longitudinal trajectories differed according to the age at first implantation (CI1), the age at second implantation (CI2), and the inter-implant interval, we fitted mixed-effects models, including time × predictor interaction terms. The interaction results for the primary outcomes are presented in Table 7, Table 8 and Table 9.

Across most outcomes, the 95% confidence intervals for the time × predictor interaction estimates included zero and the FDR-adjusted q-values were not statistically significant, indicating no clear evidence that the age at CI1, the age at CI2, or the inter-implant interval systematically modified the rate of change over follow-up in this small cohort. The only nominal association observed before the multiplicity correction was for unilateral CI2 speech recognition: an older age at CI2 was associated with a smaller improvement over follow-up in unilateral speech recognition at 60 dB (Δ change over 12 months per +1 year: −8.48 percentage points; 95% CI: −16.51 to −0.46; *p* = 0.039), although this did not remain significant after the FDR adjustment (q = 0.59) (Table 8). No corresponding effect was observed for bilateral speech recognition, which showed ceiling-level performance across timepoints (Table 3) and no evidence of effect modification by age or interval (Table 7, Table 8 and Table 9). For the vestibular endpoints (vHIT gain and asymmetry), the interaction estimates were small with wide confidence intervals, and no consistent pattern of effect modification by the age at CI1, the age at CI2, or the inter-implant interval was detected. The SSQ total similarly showed no clear evidence of effect modification (Table 7, Table 8 and Table 9).

## 4. Discussion

In this case series, we enrolled children undergoing sequential bilateral cochlear implantation. We observed a clinically meaningful improvement in CI2 unilateral speech recognition and the speech-in-noise performance over the first postoperative year, while bilateral word recognition remained consistently high. Vestibular measures were largely stable, and patient-reported outcomes (SSQ) suggested modest changes over time. Taken together, these findings preliminarily support the concept that patients could acquire substantial functional benefit from a second implant, particularly in tasks that depend on the second ear’s contribution and on listening in noise.

The most interesting result was the trajectory of unilateral CI2 speech recognition at 60 dB, which we considered as the conversational/comfortable hearing stimulus intensity. The median WRS was 0% at 3 months and increased steadily to 60% by 12 months, with widening performance ranges over time. A similar improvement has been reported previously in the literature [17,44,45]. Clinically, this pattern is consistent with a learning and adaptation process following activation of the second implant: early performance is often limited by the necessity for device familiarisation, integration of a novel electric input, and auditory training demands. These improvements are likely influenced by consistent, structured postoperative speech rehabilitation performed by all the included patients. [46]. Moreover, gains may reflect progressive hearing training and cortical adaptation [47]. Importantly, the interquartile range and min–max values indicate substantial inter-individual variability. This finding suggests that some children achieve a rapid and high performance, while others improve more slowly. This could be relevant for counselling families and planning rehabilitation.

In contrast, bilateral speech recognition at 60 dB was high already at baseline and remained near ceiling across follow-up. This likely reflects that, even before CI2, children had developed strong speech recognition with CI1 and could perform well under favourable listening conditions [48]. The ceiling effect also implies that this specific test condition may be insensitive to incremental changes attributable to the second implant and underscores the importance of including more demanding outcomes when assessing functional benefits of bilateral stimulation, such as listening in noise and spatial configurations.

Consistent with this, the speech-in-noise performance (SRT50) improved over time. In the FF configuration, the median SRT50 decreased from 8.1 dB at enrolment to 1.5 dB at 12 months (with values extending into negative SNRs), indicating an improved ability to understand speech at lower signal-to-noise ratios. Similar improvements were observed in the lateralised configurations (F1C and F2IC), with the lowest median SRT50 at 12 months in F2IC. These findings preliminarily align with the clinical expectation that bilateral implantation can provide benefits in complex acoustic environments through improved access to binaural cues, such as head shadow and spatial release from masking. Nonetheless, the magnitude of benefit varies among individuals and listening configurations, even in patients where the inter-implant interval largely extends beyond the typically recommended 12–24 months, in agreement with Kleijbergen et al. [17].

In time-only mixed-effects models, we detected a significant global effect of time for CI2 unilateral speech recognition at 60 dB and for SRT50 in the FF and F1C configurations after FDR correction, supporting a robust improvement over follow-up in this preliminary analysis. SRT50 in the F2IC configuration showed a nominal time effect that did not remain significant after a multiplicity adjustment, likely reflecting a higher variability and limited power. In contrast, bilateral speech recognition at 60 dB did not show a global time effect, consistent with the ceiling-level performance under this favourable condition. The SSQ domain and total scores demonstrated small numerical changes over time, but no statistically significant global time effects after the FDR correction. Finally, the vHIT gain and asymmetry did not show significant change over the available follow-up.

The PTA4 values were very high across all timepoints. PTA4 represents residual unaided thresholds, so a lack of improvement is expected and does not contradict the functional gains observed in speech measures; rather, it reflects limited residual hearing and emphasises that speech outcomes are the most clinically informative measures in this cohort.

The risk of vestibular impairment after cochlear implantation has been reported as 0–77% [30]. The vestibular outcome in CI candidates remains a topic of debate, and the vestibular assessment of these patients is paramount [33]. However, in this paediatric preliminary cohort, the group-level vestibular findings obtained with vHIT were broadly stable. The S.V.E.P. sensory analysis (visual, vestibular, and somatosensory contributions) showed only small changes from enrolment to activation and 6 months, which are in line with growth-related changes. This supports the hypothesis that the balance control strategy did not undergo major shifts during the early postoperative period. Furthermore, the vHIT measures suggested a reduction in median asymmetry and an increase in median gain on the CI2 side at 6 months. This finding partially aligns with what was reported by Licameli et al., who reported high rates of vestibular preservation after surgery [29]. Our data support the report of Nassif et al., who reported no difference between patients with bilateral CIs and healthy subjects over the long term [31]. Nonetheless, the absence of alterations in the vHIT results does not completely exclude subtle otolithic or low-frequency vestibular dysfunction, even in the presence of normal posturography.

While Guan et al., Devroede et al., and Wagner et al. [37,49,50] reported alterations in caloric tests and VEMPs alongside stable vHIT results, a recent study by Vibert et al. [30] (Vibert 2023) highlighted the absence of an association between caloric and vHIT results and the reported vestibular symptoms in the long-term follow-up. These authors described only a tendency of being more symptomatic at DHI at a higher implantation age. Conversely, our preliminary findings show that patients with a higher implantation age maintained normal static posturography parameters and normal vHIT results. The same results were also observed despite a long interval between the first and second implantations.

All the patients included in the present analysis received a perimodiolar array, and no complications were reported. This could exclude possible influences to hearing and vestibular function, which could be affected by an alteration in array positioning [51].

A limit of this study is that caloric testing and VEMPs were not measured in our cohort; however, any clinically relevant low-frequency vestibular impairment would likely have resulted in posturographic alterations, which were not observed in our patients. Vestibulospinal function, in fact, appears relatively preserved, whether due to pre-existing or post-surgical vestibular modifications.

The assessment of quality of life with questionnaires has already revealed a relevant role of hearing loss in children in previous studies [52]. In our preliminary analysis, the SSQ results were satisfactory in the postoperative early phase. Furthermore, our data suggest small changes over time. The SSQ total scores were similar at enrolment and 6 months, with a modest increase at 12 months. SSQ speech and quality showed increases by 12 months, whereas SSQ spatial decreased at 6 months and remained similar at 12 months. An increase in SSQ reporting was previously reported by Sparreboom et al. [53]. The domain-specific pattern observed in our sample may reflect differences in how children perceive listening abilities across contexts: speech understanding may improve earlier and be more noticeable. Conversely, spatial hearing is more complex and may require longer adaptation, specific experiences, and maturation of binaural processing. Importantly, the SSQ patterns suggest that the perceived benefit may emerge gradually and may not be uniform across domains, which supports individualised counselling and rehabilitation planning.

In exploratory mixed-model analyses, we did not detect that the age at CI1, the age at CI2, or the inter-implant interval systematically modified the rate of change over follow-up across the primary outcomes, aligning with previous literature [13,48,54]. In our sample, these findings could have been influenced by the small sample size or the high level of inter-individual variability. Nonetheless, similar findings were reported by Jang et al. [11], supporting the positioning of sequential bilateral CIs in good CI1 performers with bilateral profound hearing loss. Vibert et al. reported the stability of findings in the long-term follow-up of vestibular function [30]. A study by Mendes et al. also described high quality of life rates in long-inter-implant-interval patients [55]. A nominal association was observed for unilateral CI2 speech recognition with the age at CI2, suggesting that an older age at CI2 might be associated with smaller gains. Previous studies reported the sequential CI as potentially beneficial in bilaterally hearing-impaired patients [35,36,56]. Moreover, several studies have reported less satisfactory hearing results in patients with longer inter-implant intervals [49,57,58].

### 4.1. Clinical Implications

These findings may have practical implications for counselling and follow-up. First, families should be informed that the unilateral performance with CI2 may be very limited early after activation, especially in the case of longer inter-implant intervals [59], reducing the usage rate of CI2 [38]. Substantial gains were observed over the first year, supporting sustained rehabilitation. Second, outcomes near ceiling in quiet should not be over-interpreted; improvements seem to be better captured by noise and spatial listening measures. Third, the variability across individuals reinforces the need for personalised monitoring and targeted therapy, rather than assuming a uniform benefit or uniform timelines. Some patients may benefit from stricter follow-up and more frequent rehabilitation, whereas other patients could benefit by looser patterns of seriated evaluation.

### 4.2. Limitations

This study is limited primarily by the small sample size and its observational design. Given the small sample size, analyses were considered exploratory and interpreted as hypothesis-generating. Several outcomes (notably bilateral speech recognition) showed ceiling effects, while unilateral CI2 speech recognition showed early floor effects, both of which reduce the sensitivity to detect predictors of change. Finally, the sensory-weight balance measures are compositional, and while descriptive results are informative, inferential modelling requires specialised approaches.

## 5. Conclusions

In this preliminary cohort of children receiving a second sequential cochlear implant, we observed improvements over time in unilateral CI2 speech recognition and the speech-in-noise performance, with stable, high bilateral speech recognition in quiet and no major group-level vestibular deterioration during early follow-up. These findings were observed even if the interval between the two CIs in our sample was longer than those reported by other reports. Exploratory analyses did not show that the implantation ages or inter-implant interval consistently modified our preliminary longitudinal trajectories. Substantial gains can be observed over the first year. Larger studies are required to confirm these preliminary findings and identify predictors of individual benefit.

## Figures and Tables

**Table 1:** Evaluation protocol for sequentially bilaterally implanted children (The “x” indicates when the tests were performed along the follow-up).

	Pure-Tone Audiometry	Speech Audiometry			Speech-in-Noise	Posturo.	vHIT	Questionnaires	Language Evaluation Battery
		CI1	CI2	CI Bilateral					
Enrol.	x	x			x	x	x	x	x
3rd mo.			x	x	x				x
6th mo.	x		x	x	x	x	x	x	x
9th mo.			x	x	x				x
12th mo.	x		x	x	x			x	x

**Table 2:** Demographic data.

ID	Sex	Aetiology	Enrolment Age (YY)	CI1 Side	CI1 Age (YY)	CI2 Age (YY)	Type of Array	Inter-Implant Interval (YY)
1	M	Unknown	13	L	2	13	Perimodiolar	10
2	M	Ototoxicity	11	L	6	11	Perimodiolar	5
3	F	Connex-26	7	L	1	7	Perimodiolar	5
4	F	Unknown	14	L	4	15	Perimodiolar	10
5	F	Unknown	10	L	2	10	Perimodiolar	8
6	M	Unknown	10	L	2	10	Perimodiolar	8
7	F	CMV	15	R	0	15	Perimodiolar	14
8	M	Unknown	9	R	8	9	Perimodiolar	1
9	F	CMV	10	L	2	10	Perimodiolar	7
10	M	Usher Sdr	12	R	2	12	Perimodiolar	9

**Table 3:** Audiological primary outcomes by timepoint.

Outcome	Timepoint	N	Median [p25, p75]	Min–Max
PTA4 (dB)	act	10	115.62 [103.75, 120.62]	93.75 to 125.00
PTA4 (dB)	1 m	10	117.50 [116.56, 120.00]	100.00 to 125.00
PTA4 (dB)	6 m	10	120.00 [117.81, 120.94]	106.25 to 125.00
PTA4 (dB)	12 m	10	120.00 [119.06, 122.19]	102.50 to 125.00
SRT50 F1C (SNR dB)	enr	10	11.90 [ 7.50, 13.65]	4.00 to 15.80
SRT50 F1C (SNR dB)	3 m	10	5.50 [ 4.35, 10.10]	3.00 to 15.00
SRT50 F1C (SNR dB)	6 m	10	3.00 [−1.75, 3.30]	−4.00 to 14.20
SRT50 F1C (SNR dB)	9 m	10	4.70 [ 0.25, 9.00]	−6.00 to 11.00
SRT50 F1C (SNR dB)	12 m	10	3.00 [−5.75, 5.90]	−11.00 to 10.80
SRT50 F2IC (SNR dB)	enr	10	7.40 [ 4.05, 10.90]	−1.00 to 13.00
SRT50 F2IC (SNR dB)	3 m	10	8.60 [ 0.20, 14.00]	−11.00 to 17.00
SRT50 F2IC (SNR dB)	6 m	10	4.00 [ 0.50, 8.15]	−10.00 to 14.40
SRT50 F2IC (SNR dB)	9 m	10	3.50 [ 1.00, 7.40]	−2.00 to 16.00
SRT50 F2IC (SNR dB)	12 m	10	0.00 [−0.75, 1.45]	−11.00 to 10.00
SRT50 FF (SNR dB)	enr	10	8.10 [ 6.70, 10.40]	5.00 to 14.80
SRT50 FF (SNR dB)	3 m	10	6.20 [ 4.00, 9.65]	−1.20 to 13.00
SRT50 FF (SNR dB)	6 m	10	3.00 [ 2.25, 3.45]	−1.00 to 11.60
SRT50 FF (SNR dB)	9 m	10	4.50 [ 1.25, 6.65]	−1.00 to 11.40
SRT50 FF (SNR dB)	12 m	10	1.50 [−3.00, 4.50]	−11.00 to 8.40
Speech Bilateral—60 dB (WRS%)	enr	10	95.0 [80.0, 100.0]	60.0 to 100.0
Speech Bilateral—60 dB (WRS%)	3 m	10	95.0 [82.5, 100.0]	40.0 to 100.0
Speech Bilateral—60 dB (WRS%)	6 m	10	100.0 [90.0, 100.0]	70.0 to 100.0
Speech Bilateral—60 dB (WRS%)	9 m	10	95.0 [90.0, 100.0]	60.0 to 100.0
Speech Bilateral—60 dB (WRS%)	12 m	10	100.0 [85.0, 100.0]	60.0 to 100.0
Speech Unilateral (CI2)—60 dB (WRS%)	3 m	10	0.0 [ 0.0, 7.5]	0.0 to 50.0
Speech Unilateral (CI2)—60 dB (WRS%)	6 m	10	15.0 [ 0.0, 55.0]	0.0 to 60.0
Speech Unilateral (CI2)—60 dB (WRS%)	9 m	10	35.0 [12.5, 75.0]	0.0 to 90.0
Speech Unilateral (CI2)—60 dB (WRS%)	12 m	10	60.0 [32.5, 87.5]	10.0 to 100.0

**Table 4:** Vestibular primary outcomes by timepoint.

Outcome	Timepoint	N	Median [p25, p75]	Min–Max
Somatosensory (%)	enr	10	0.342 [0.315, 0.349]	0.250 to 0.383
Somatosensory (%)	act	10	0.345 [0.310, 0.349]	0.300 to 0.380
Somatosensory (%)	6 m	10	0.342 [0.308, 0.379]	0.278 to 0.425
Vestibular (%)	enr	10	0.270 [0.252, 0.297]	0.210 to 0.370
Vestibular (%)	act	10	0.260 [0.251, 0.298]	0.160 to 0.340
Vestibular (%)	6 m	10	0.259 [0.245, 0.276]	0.180 to 0.340
Visual (%)	enr	10	0.385 [0.362, 0.414]	0.308 to 0.524
Visual (%)	act	10	0.391 [0.352, 0.406]	0.332 to 0.529
Visual (%)	6 m	10	0.385 [0.353, 0.434]	0.313 to 0.519
vHIT asymmetry (%)	enr	8	0.215 [0.048, 0.428]	0.040 to 0.580
vHIT asymmetry (%)	6 m	9	0.110 [0.060, 0.270]	0.010 to 0.450
vHIT gain (CI2 side)	enr	8	0.740 [0.538, 0.870]	0.360 to 1.070
vHIT gain (CI2 side)	6 m	9	0.830 [0.640, 0.960]	0.250 to 1.030

**Table 5:** SSQ domain and total scores by timepoint.

Outcome	Timepoint	N	Median [p25, p75]	Min–Max
SSQ Quality	enr	10	6.72 [6.44, 7.28]	4.35 to 8.40
SSQ Quality	6 m	10	6.80 [5.99, 7.27]	4.80 to 9.25
SSQ Quality	12 m	10	7.44 [6.68, 7.77]	6.15 to 8.30
SSQ Spatial	enr	10	6.00 [4.08, 6.54]	2.83 to 7.69
SSQ Spatial	6 m	10	5.37 [4.67, 5.89]	3.50 to 9.00
SSQ Spatial	12 m	10	5.43 [5.18, 6.09]	4.38 to 7.92
SSQ Speech	enr	10	6.25 [4.90, 7.71]	3.40 to 8.40
SSQ Speech	6 m	10	6.18 [5.96, 6.99]	3.88 to 8.85
SSQ Speech	12 m	10	7.28 [6.73, 7.79]	5.30 to 8.55
SSQ Total	enr	10	6.39 [5.19, 6.87]	3.84 to 8.01
SSQ Total	6 m	10	6.12 [5.58, 6.25]	4.40 to 9.03
SSQ Total	12 m	10	6.66 [6.42, 6.78]	5.64 to 8.21

**Table 6:** Global time effect (mixed models): do outcomes change over time?

Outcome	Timepoints	Global p (LRT)	FDR q
Speech unilateral 60 dB (WRS%)	4	0.0000	0.0001
Speech bilateral 60 dB (WRS%)	5	0.6176	0.6176
SRT50 F1C (SNR dB)	5	0.0000	0.0000
SRT50 FF (SNR dB)	5	0.0003	0.0014
SRT50 F2IC (SNR dB)	5	0.0240	0.0721
PTA4 (dB)	4	0.0463	0.1111
SSQ quality	3	0.1678	0.2734
SSQ spatial	3	0.5208	0.5681
SSQ speech	3	0.1823	0.2734
SSQ total	3	0.1743	0.2734
vHIT asymmetry	2	0.3489	0.4653
vHIT gain (CI2 side)	2	0.4956	0.5681

**Table 7:** Interactions of time × age at CI1 (years).

Outcome	Follow-Up Window	Δ Slope per +1 Year	95% CI	Δ Change over Follow-Up per +1 Year	95% CI	*p*	FDR q
Speech bilateral 60 dB HL (WRS%)	0–12 months	−0.0113	[−0.3866, 0.3639]	−0.1361	[−4.6388, 4.3666]	0.9515	0.9515
Speech unilateral 60 dB HL (WRS%)	0–12 months	−0.172	[−0.9363, 0.5923]	−2.0643	[−11.2362, 7.1076]	0.6483	0.8104
SRT50—frontal noise (SNR dB)	0–12 months	0.0278	[−0.0868, 0.1425]	0.3339	[−1.0421, 1.7099]	0.6261	0.8104
vHIT asymmetry	0–6 months	0.0073	[−0.0075, 0.0222]	0.044	[−0.0451, 0.133]	0.3055	0.6547
vHIT gain (CI2 side)	0–6 months	−0.0021	[−0.0115, 0.0073]	−0.0127	[−0.0691, 0.0437]	0.6049	0.8104
SSQ total	0–12 months	0.016	[−0.0105, 0.0425]	0.1918	[−0.1264, 0.51]	0.2215	0.5537

**Table 8:** Interactions of time × age at CI2 (years).

Outcome	Follow-Up Window	Δ Slope per +1 Year	95% CI	Δ Change over Follow-Up per +1 Year	95% CI	*p*	FDR q
Speech bilateral 60 dB HL (WRS%)	0–12 months	0.0034	[−0.3502, 0.3569]	0.0403	[−4.202, 4.2825]	0.9848	0.9893
Speech unilateral 60 dB HL (WRS%)	0–12 months	−0.7069	[−1.3759, −0.0379]	−8.4832	[−16.5113, −0.4552]	0.0391	0.5865
SRT50—frontal noise (SNR dB)	0–12 months	0.0134	[−0.0949, 0.1216]	0.1603	[−1.1391, 1.4597]	0.8042	0.9893
vHIT asymmetry	0–6 months	0.0016	[−0.0139, 0.0172]	0.0098	[−0.0837, 0.1033]	0.8237	0.9893
vHIT gain (CI2 side)	0–6 months	0.0009	[−0.009, 0.0107]	0.0052	[−0.0538, 0.0643]	0.8359	0.9893
SSQ total	0–12 months	−0.0052	[−0.0312, 0.0207]	−0.0627	[−0.374, 0.2486]	0.6773	0.9893

**Table 9:** Time × inter-implant interval (years).

Outcome	Follow-Up Window	Δ Slope per +1 Year	95% CI	Δ Change over Follow-Up per +1 Year	95% CI	*p*	FDR q
Speech bilateral 60 dB HL (WRS%)	0–12 months	0.0018	[−0.256, 0.2596]	0.0214	[−3.0718, 3.1147]	0.9889	0.9889
Speech unilateral 60 dB HL (WRS%)	0–12 months	−0.2822	[−0.7978, 0.2334]	−3.3863	[−9.5734, 2.8009]	0.2718	0.8153
SRT50—frontal noise (SNR dB)	0–12 months	−0.003	[−0.082, 0.076]	−0.0361	[−0.9843, 0.9121]	0.9389	0.9889
vHIT asymmetry	0–6 months	−0.0025	[−0.0127, 0.0077]	−0.0152	[−0.0764, 0.0459]	0.5999	0.9889
vHIT gain (CI2 side)	0–6 months	0.0008	[−0.0055, 0.007]	0.0046	[−0.0331, 0.0423]	0.7771	0.9889
SSQ total	0–12 months	−0.0106	[−0.0288, 0.0077]	−0.1267	[−0.346, 0.0925]	0.2403	0.8153

## Data Availability

The data presented in this study are available from the corresponding author upon request. The data are not publicly available due to privacy restrictions.

## Abbreviations

The following abbreviations are used in this manuscript:

CI	Cochlear Implant
SSQ	Speech, Spatial and Qualities of Hearing Scale for Children
VEMPs	Vestibular Evoked Myogenic Potentials
vHIT	Video Head Impulse Test
